# ‘I Can Tell You It’s a Bit of a Gamble’: A Qualitative Analysis of How People Who Engage in Gaming and Gambling Understand a Link Between These Two Behaviours

**DOI:** 10.1007/s10899-023-10275-2

**Published:** 2023-12-15

**Authors:** Łukasz Wieczorek, M. Bujalski, K. Dąbrowska

**Affiliations:** 1 Department of Studies on Alcoholism and Drug Dependence, Institute of Psychiatry and Neurology, Sobieskiego 9 Street, 02-957 Warsaw, Poland; 2 Institute of Applied Social Sciences, University of Warsaw, Nowy Świat 69 Street, 00-046 Warsaw, Poland

**Keywords:** Video games, Gambling, Gambling disorders, Risk perception

## Abstract

This article explores the attention given to potential motives and determinants of the transition process from video games to gambling. Forty individual interviews were conducted among active video game players (n = 20), and people diagnosed with a gambling disorder who had the experience of playing video games (n = 20). A qualitative thematic analysis was employed to explore the collected empirical data. The range of factors considered responsible for the transition from playing video games to gambling included experiencing similar emotional states, the presence of gambling in video games, advertising of gambling companies in video games, loot boxes. The awareness of factors associated with the development of gambling disorders among video game players has an important role in informing prevention policies in the rapidly changing video gaming and gambling market. Decision-makers should introduce effective regulation of the use of gambling components in video games to protect gamers against the gambling-related harm.

## Introduction

Research have shown that gaming may serve as a pathway to an increased risk of problem gambling (Griffiths, 2002; Macey & Hamari, 2018; Molde et al., 2019). In this regard, the structural similarities between gaming and electronic gambling are considered a major feature (Fisher & Griffiths, 1995, Johansson & Götestam, 2004, Wood et al., 2004), with following various social benefits enjoyed by skillful players (Griffiths & Wood, 2000) and reduced individual sense of control (Gupta & Derevensky, 1996).

The world of video games has changed dramatically in recent decades, switching from simple arcades games to sophisticated online games, in which players may cooperate or compete with other players or with AI. Gaming spaces have become dispersed, being no longer located in video game arcades or at homes. Currently games can be played virtually everywhere using various electronic mobile devices (Hamari et al., 2017; Macey & Hamari, 2019). Business models such as free-to-play and social network gaming have effectively introduced gambling-like elements into video games. Additionally, the use of virtual currencies in video games to pay for gambling-like activities have become a more and more common, which resulted in the increased the uncertainty about the nature of the game among the players (Gainsbury et al., 2016; King et al., 2010). In fact, for some, playing video games has become a way to make money (Griffiths, 2017).

The awareness of the link between gaming and gambling have been recently addressed in several scientific studies. However, rather mixed results have been deliverd. Some studies showed a link between playing video games and increased participation in gambling (Gainsbury et al., 2016, Kim et al., 2015, McBride & Derevensky, 2017, Wood et al., 2004), while other were not able to definitely confirm this link (Delfabbro et al., 2009, Forrest et al., 2016, King et al., 2012). For example, the results of the study by Gainsbury et al. (2016) showed that in the opinion of majority of surveyed individuals, *social casino games* were considered similar to gambling in terms of the visuals and gaming experience. More than half of the players felt that the excitement of winning was similar to that of gambling. About one-fifth of *social casino game* players claimed that their experience in playing these types of games increase their chances for gambling success. A study by McBride and Derevensky (2017) compared gambling behaviour among gamers and non-gamers as well as gaming behaviour among gamers and non-gamers. Their results suggested that gamblers were more likely to play video games than non-gamblers, and video game players were more likely to gamble than non-players. Zendle and Cairns (2018) found evidence for a link between the amount of money spent on loot boxes and the severity of problem gambling among gamers. This link was stronger than a link between problem gambling and purchasing other in-game items with real-world money, suggesting that the gambling-like features of loot boxes had significant impact on the observed results. On the other hand, Forrest et al. (2016) found that the frequency of playing video games does not significantly correlate with the frequency of gambling. Results of study by Macey and Hamari (2018) showed no robust associations between playing video games and gambling, but problem gaming was found to have a significant, however small negative association with both problem gambling and gambling in general. In turn, Delfabbro and King (2021) concluded that there is only a slight evidence that video-games may lead to gambling and advocated for further research investigating factors that may facilitate the transition from playing video games to gambling.

Considering the above, this study was undertaken to explore the attention given to potential motives and determinants of the transition process from video games to gambling. Specifically, we sought to (1) examine whether gamers and individuals diagnosed with gambling disorder are aware of the similarities between video games and gambling; and (2) investigate whether the similarities of video games and gaming are perceived as factors that facilitate transition to gambling.

## Methods and Data

### Sample Recruitment

The reported study was conducted in Poland between April and July 2020. A qualitative approach was employed, allowing for exploration of meanings, knowledges, and experiences of gaming and gambling issues. Purposive sampling was used to recruit study participants.

A total of 40 individual interviews were conducted including 20 interviews with active video game players, and 20 interviews with people with gambling disorders who had an experience of long-term engagement in video gaming. Respondents provided their subjective opinion and shared their own way of understanding of the link between gaming and gambling. The data collected from these two groups allowed for a more in-depth investigation to identify various factors that might not have been otherwise explored if only one group were considered, which translates into improved comprehensiveness of the data (Barbour, 2001; Braun & Clarke, 2006; Mays & Pope, 2000).

Respondents from the gambling disorder group were recruited in addiction treatment facilities in the Warsaw area. At each facility its manager was contacted to assist with recruitment process. Due to the pandemic restrictions, facility managers were asked to hand on the information about the study to the therapists, whose task was to invite patients meeting the inclusion criteria to the interviews. Patients who agreed to participate shared their telephone number. Video game players were recruited through thematic groups on social media sites (Facebook, Twitter, etc.) and online forums. Additionally, the snowball sampling method was used for completing the recruitment procedure. Members of research team contacted all participants personally to complete the interview by telephone or by on-line video communicators.

### Inclusion Criteria

The inclusion criteria for active video game players were age (16–30 years old) and playing online and/or offline video games (including Pay2Win games) at least once in the past week. Pay to Win games were defined as a common type of video game design where players can pay to accelerate their progress. The frequency and value of these transactions are unlimited, and linked to the players’ competitiveness or progress within the game (Steinmetz et al., 2022).

Some of the participants of this group had previous or current experience with gambling, but this was not a prerequisite for participation in the study. The age restriction for video game players was based on the fact that people aged 16–30 commonly engage in this kind of activities (Polish Gamers Observatory, 2019) while also might already have some experience with gambling. In contrast, the inclusion criteria for individuals with a gambling disorder were being 18 years old or older, being diagnosed with a gambling disorder, undergoing treatment in a specialized addiction treatment facility and having played online and offline computer games (including Pay2Win games) at least once in the past week. Both video game players and people with gambling disorders were asked to provide their informed consent to participate in the study.

### Sample Characteristics

Most respondents in the study (n = 32) were men. In the group of video game players, one in four respondents was a woman. In contrast, there was only one woman in the gambling disorder group, while two participants did not provide information on their gender. The mean age of interviewed video game players was 23 years old (SD = 7). In contrast, respondents with gambling disorder were older, with the mean age of 30 years old (SD = 3). Members of both groups had their secondary or university education completed, while only two people from each group had primary or vocational education. Most of video game players (n = 17) lived in large towns (more than 100,000 inhabitants). In the group of people with gaming disorders, most lived in large towns (n = 12) while every third person (n = 5) lived in a small town. One in two individuals in the video game group had a job, one in three (n = 6) was studying and one in seven (n = 3) were unemployed. In the gambling disorder group, nearly a half were unemployed, one in three (n = 6) had a job and one in five (n = 3) were studying (Table 1).

**Table 1:** Sociodemographic characteristics of the respondents

Category		Video game players	People with a gambling disorder
		N	N
Gender	Male	15	17
	Female	5	1
Age	18–25	20	5
	26 and over	0	13
Education	Primary or vocational	2	2
	Secondary	13	9
	University	5	7
Place of residence	Town with more than 100,000 inhabitants	17	12
	Town with 50,000–100,000 inhabitants	1	1
	Town with under 50,000 inhabitants	2	5
Occupational status	Employed	11	6
	Unemployed	3	8
	Student	6	3

### Research Tools

Interviews were semi-structured and included the same set of questions for the two groups of respondents. Following topics were covered: video games of choice, preferred type of game, motives for choosing them, and the amount of money spent on game upgrades. Another set of questions concerned the experiences of gambling—types of games, motives for gambling, spending money. Next, we asked about perceived similarities and differences between video games and gambling, as well as about the elements of video games that might encourage gambling, reasons of gambling, accessibility to video games and gambling games, and the perceived risk of developing addiction to these two behaviours. The interviews lasted ca. 40 min. All interviews were audio-recorded, coded to ensure anonymity and transcribed verbatim. The final dataset contained material from both groups thus providing two different perspectives on the phenomenon of the relation of playing video games and gambling as well as the accounts on the risk of developing a gambling disorder in gamers.

### Data Analysis

When coding the data, we attended to the specific characteristics of gamers and gamblers regarding the above issues. Informed by the approach presented by Miles and Huberman (2000), we employed two levels of thematic coding to analyze the data. At the initial level, individual sentences and quotes were assigned codes created during the ongoing analysis (e.g. gambling for money, playing video games to keep in touch with friends). At the subsequent level, individual codes were assigned to broader thematic groups inspired by the matrix cited by Macey and Hamari (2018) as well as to new emergent categories. The three researchers (ŁW, KD and MB) agreed a coding frame. Next, the material was thematically coded by two authors (ŁW and KD). Disagreements about coding or its relevance as well as identification of thematic categories were referred to a third author (MB). The analysis was conducted with ATLAS.ti software.

### Ethical Issues

The study was approved by the Bioethical Commission at the Institute of Psychiatry and Neurology (Warsaw, Poland), with decision no. 12/2019. Participation in the study was voluntary. The participants were provided with information about the study characteristics (introduction to the study, objectives and methods, estimated duration of the study and assurances about the confidentiality of the data collected). In order to ensure anonymity, the interviewed individuals did not sign a consent form to participate in the study. Instead, the interviewers obtained their verbal consent to participate and filled in statements about the respondent’s conscious agreement to participate in the study. The interviews were coded with a number indicating the group, gender and the record number; personal data were not collected. The respondents did not receive any remuneration for participating in the study.

## Results

According to available research data, the process of shifting from playing video games to gambling can be generally categorized into three categories recalled by Macey and Hamari (2018), that is: structural similarities between video game and gambling, social benefits or losses, and misconceptions of control. In our study, we were able to confirm the awareness of the above categories as well as distinguish two additional: experiencing similar emotions and sharing a need of escaping from reality in both groups. These highlight new potential dimensions of gaming-gambling correspondence—emotional gratification and the creation of one’s isolated world.

### Structure of the Video and Gambling Games

This dimension is composed of two elements: competition and desire to win, and the aspect of randomness. One of the major similarities between video games and gambling was related to the need of competition and the desire to win. Both video game players and people who played gambling games wanted to compete and win against other players and the structure of these games allows them to satisfy these needs. Therefore, we have included competition and the desire to win in the dimension related to the structure of games. Both video games and gambling games provide players with the ability to meet these needs. Video games require the player to compete with others which may reinforce the desire to win. Apparently, the same can be found in gambling games.

**Table:** 

Competition and the will to win	Video gaming group: *there is a strong competitive element in both cases. Note that most computer games, video games that utilize the gambling mechanics, such as loot boxes, boosters and so on, are those that have a strong competitive edge. And it’s the same with gambling. After all, when you’re sitting in an e-casino, well, you’re playing at a table with some other people and you want to win.* (VGMO280520203^a^)
	Gambling: *The common thing here is a desire to win. The will to win. In gambling you win money and in computer games you can either play for money or not.* (GDAL160820201)

^a^The method of coding: VG—video game player; GD—people with gambling disorder; XXXXXXXX—date when the interview was conducted; Y—number of the interview conducted on the same day

Another common characteristic of video games and gambling highlighted by our respondents was an aspect of randomness. For video game players, lootboxes were indicated as an example of such randomness which might encourage gambling behaviours. Players associated the aspect of randomness with the unknown content of the loot box, linked to the uncertainty experienced when playing a gambling game. The experience of players shows that it is usually low-value items that can be obtained with money, but sometimes one can get extremely rare item which makes it highly valuable. Gambling features can be also found integrated with the structure of game. For example, in popular FPS counter strike, one of the most played games globally, players can take part in a roulette, in which they pay a small amount of money but do not know what they will receive in turn. One can win items that make the game more attractive and appealing. According to players, the nature of counter strike roulette is similar to classic gambling roulette. Moreover, this feature is perceived as associated with gambling on semantic level as the meaning of the term *roulette* makes an obvious reference to gambling.

**Table:** 

Randomness	Video game player: *I can tell you that it’s a bit of a gamble. For example, there’s a game called Counter-Strike, and there are skins for those weapons in Counter-Strike, and they don’t change anything except their appearance, but there are some sites where you have kind of roulette, where you pay for example, five Zloty or something, and it draws you a random skin. It’s like gambling for me, because you don’t know what you’re buying, but you spend money still.* **(**VGMD200620201)

In addition, gambling games are deployed within the real-world gaming environment comprising of various tournaments and online-streamed matches. Here, players can bet on the gameplay, using their items or real money. This is perceived by players as a form of placing bets with bookmakers just like in football matches.

**Table:** 

Gambling as an element of video games	Video game player: *there was an option to bet on e-sports matches, or those of professional Counter-Strike players. Betting on matches just like in football, it’s the same in Counter-Strike, only you bet with your items or money. I started playing with two dollars, betting on matches, and eventually I made two or three hundred dollars.* (VGMT150620201)

Interviewed individuals with gambling disorder were highly aware of gambling advertising embedded in the video games. As they argued, for people addicted to gambling advertisements can have triggering effect and may encouraged people with gambling disorders to take further risks. It was also emphasized that people who are not addicted do not tend to recognize advertising in video games as an element which encourage gambling, because they are not sensitive to triggers that can otherwise disrupt abstinence in individuals who already experience gambling addiction.

**Table:** 

Gaming advertising in video games	A person with a gambling disorder: *in FIFA, which is a game that has a license for the names of the clubs and their players’ outfits, all football shirts or the vast majority of them have the bookmakers’ adverts on them. In some way this is something that encourage someone to gamble. But I would also make a distinction here, because in the case of a gambling addict like me can see these ads and I know they are a trigger, right? That is, a potential threat that can trigger an urge to gamble. But someone who is not addicted to gambling will also notice this advertisement, will be aware of what it is, but will not feel encouraged to gamble.* (GDSD010720201)

### Social Benefits and Losses

Within this dimension, respondents emphasized the possibility of financial benefits and recognized the risks associated with the loss of funds. The financial benefit derived from engaging in both video games and gambling lies at the intersection of these to activities. Video game players compete for money by engaging in e-sports matches, they also sell their profiles developed during playing sessions. They collect items, skins, and elements of equipment to use as a source of income. Video game players can also make profits by playing gambling games embedded in video games. The virtual funds received as a reward can be spent for example on their game character development.

**Table:** 

Getting material benefits	Video game player: *for example, we used to create a character which would be quite good, like in the top ten. And there are players, for example, who don’t want to play from the beginning of the game and do everything in it, so they make you an offer that they will buy your character for a few dozen Zloty or so.* (VGJM270520201)

In addition to the financial losses experienced while playing video games, respondents also talked about the costs spent on buying premium accounts, characters development, and loot boxes. However, in case of gambling games, financial losses occurred when they lost. Individuals with gambling disorders admitted that the more they played, the higher financial losses were generated. The same would occur when they engaged in video games, as once they were more involved in the game, they spent more money on it.

**Table:** 

Financial losses	A person with a gambling disorder: *the financial losses were very similar in both cases. For example, buying these premium accounts, collecting more points to get to a higher level, trying to get a better item. It looked like you were buying cheaper, but you ended up spending a fortune.* (GDMO070720203)

### Misconceptions of Control

Among the factors associated with misconceptions of control one can distinguish the desire to play, the loss of the sense of control, and the cognitive bias related to receiving positive results in gambling games placed in video game scenario. Video games are similar to gambling games as they produce the desire to play. In video games, this may occur when players encounter a difficult moment and are not able to go any further. Then they spend more time playing as they try to overcome the difficulty. In fact, this makes the game more engaging and absorbing.

**Table:** 

Playing a return game	Video game players: *when it comes to single-player type games, like kind of offline strategic games—let’s say, there’s a difficult mission and I’ve failed a couple of time. I’ll definitely want to play to get succeed next time. Like it is kind a gamer syndrome or something like that.* (VGKM110520201)

Individuals with gambling disorders were highly aware of the addictiveness of video gaming and gambling games. It was emphasized that both types of games are easily available online, which makes them highly accessible and can be linked to the risk of losing control over the game and over the time spent playing. Respondents admitted that they spend their time not only playing the game, but also thinking about a future game session, including possible strategies how they should conduct future gameplay or their character development scenarios.

**Table:** 

Risk of addiction	Person with a gambling disorder: *both video and gambling games can be addictive. The distance [between the two] is short, especially in the context of young people and the internet as they spend so much time online, with their phone in their hand, playing games on their phone or on a console.* (GDSD010720201)
Loss of control	A person with a gambling disorder: *I used to waste a lot of time on it; when I wasn’t playing games, I was constantly thinking about what I was going to download, what I was going to do, how to expand the character in game. I was thinking about the games all the time. When I wasn’t playing, for example, I had intrusive thoughts about it all the time. And the same thing I can say about gambling. At home, I used to think about what matches to bet on.* (GDMO070720203)

According to people with gambling disorders, being successful in a video game may contribute to engaging in gambling. This is particularly the case when players achieve winnings in gambling game embedded in a video game because they might want to try gambling in a real life to test themselves or to compare the potential results with those obtained in a video game.

**Table:** 

Positive results in a video game	Person with gambling disorder: *in computer games you win something when you get lucky, so in gambling games. And I think a player can be drawn to that. For example, they may think “if I’m lucky here, I’ll get lucky in these gambling games too”.* (GDMO280520201)

### Experienced Emotions

The emotions experienced when playing video games and gambling games tend to be similar. This was particularly experienced by individuals with gambling disorders who were also engaging in video games. They were able to recognize emotions that would accompany them during gambling activities when playing video games. Video game players were less likely to mention that the emotions that occur when playing video games can be similar to those of gambling. This may be due the fact that video game players usually do not experience high losses because they play at lower stakes.

**Table:** 

Similar emotions	Person with a gambling disorder: *a person with a gambling addiction has certain mental disorders and may also experience similar feelings while playing video games*. (GDSD010720201)

### Escape from Reality

Escape from reality may be an additional dimension on the catalogue of similarities between video games and gambling. This feature was often mentioned by gamblers, according to which, playing video games is a form of escape from reality just as gambling. When they gambled, they didn’t think about anything else; about their problems, emotions and other things that were problematic in their lives. The same was claimed in relation to video games as people can spend hours on playing them, remaining out of touch with reality, not thinking about anything except the game itself.

**Table:** 

Escape from reality	Person with gambling disorder: *I was playing gambling games and I wasn’t thinking about anything, anywhere, any problems or emotions, or any different things that were affecting me. Gambling offered me this escape. And I guess that in video games it’s also strongly addictive feature; you can play for a couple of hours or many more hours and not really think about anything else but the game. So it also allows for that kind of detachment from reality.* (GDWB110320201)

## Discussion

In recent years, the link between video gaming and gambling has been drawing continuous attention of addiction science scholars (Griffiths, 2017), therefore the aim of the present study was to explore the perception of factors associated with the transition from playing video games to gambling, among video game players and individuals with gambling disorder. The current study sought to follow the characteristics presented in other studies conducted in the field in gaming and gambling problems (i.e. Delfabbro & King, 2021; Macey & Hamari, 2018; Zendle & Cairns, 2018) to show how gamers and individuals with gambling disorder tend to identify various factors, which might contribute to the process of developing addictive behaviours.

To initially structure the factors, we used the matrix cited by Macey and Hamari (2018), which distinguishes following categories: structural similarities between video games and gambling, social benefits derived from playing video games and gambling, and characteristics of these games related to illusory sense of control. The results of our study allowed us to broad the above matrix with two dimensions: experiencing similar emotions when playing video game and gambling and the motive of escaping from reality previously emphasised by King et al. (2012).

Among the group of factors that can be categorized as structural similarities between video games and gambling, respondents discussed the opportunity to compete, desire to win and the aspect of randomness. Research shows that video game players wish to experience the same emotions when playing gambling games, e.g., excitement, a sense of fun, competition (Teichert et al., 2017). The pursuit of competitive opportunities and winning present in video games can encourage people to turn to gambling games that include competitive elements, such as playing roulette or poker. In turn, the aspect of randomness in video games can be easily found in loot boxes. There are many similarities between loot boxes and gambling, including their random content and the requirement to pay to gain the access to it (DeCamp, 2021). These similarities may cause individuals with gambling disorders to spend large amounts of money to buy loot boxes in video games, just as if they were gambling. This is due to the fact that loot boxes base on randomness causing various emotional states (Zendle & Cairns, 2018). Both video game players and people with a gambling disorder were highly aware of the presence of gambling in video games. It was emphasised that many video games offer microtransactions and various options to increase the chance of winning with real money, which might encourage future gambling behaviours. Some games (e.g. EVE Online), allow for the opportunity to use the game’s virtual currency to place real sports bets or esports matches (Macey, 2021). Thus, the transition from virtual gambling in video games to real gambling using game-specific currencies can have direct detrimental effects on players both in terms of in-game and real-world consequences. Advertisements of gambling companies in video games can also be a risk factor of developing gambling problems and should be scrutinized in-depth in further research on the topic.

Among the risk factors associated with the social benefits of gaming, respondents emphasised the opportunity to earn money. Some video game players recognize the financial benefits of playing to be the additional cue for further engagement. Moreover, for those who gamble, the desire to get extra money can be an important motive for playing (Mathieu et al., 2020). However, people with gambling disorders also recognize the risk of financial loss in video games, something that video game players seem to overlook. Barely controlled expenses for a game character development or upgrading character’s equipment, as well as betting on e-sports matches, can be easily recognized by people with gambling disorders who experienced severe losses from gambling but are largely ignored by video game players.

Our study also confirms that misconceptions related to the sense of control may trigger switching from playing video games to gambling. An example of this type of behaviour is when an individual succeeding in a gambling game that is a part of video game scenario starts seeking the opportunity to test him or herself in a real-life situation.

As various studies have shown, playing video games may be associated with having false expectations about the degree of control over gambling, especially when it comes to online forms of gambling. Since many video games require skill and strategy, some players may believe that by getting experience in video games they become skilled enough to succeed in gambling. Players may also share such beliefs when they gamble without betting real money. This is particularly deceitful from the gaming operators as some games may misrepresent the chances of winning, for example online casinos may offer ‘free games’ which have been shown to overstate the chances of winning (King et al., 2012). Online gambling should be of particular focus here as research show that the online environment promotes risk-taking and impaired self-control (Gainsbury et al., 2016).

Video games and gambling may also satisfy similar emotional needs and/or provide relaxation. Therefore, people who play video games may also engage in gambling as an alternative source of stimulation, excitement or in need to relieve the experience of negative emotional states. Individuals with emotional difficulties may engage in behaviours such as playing video or gambling games to seek emotional regulation (McBride & Derevensky, 2017). Therefore, gambling or playing video games becomes an attractive activity because it satisfies the need for excitement or relaxation and provides a way to escape from reality (King et al., 2012).

All in all, our study and provides additional information on the specific factors which may contribute to the transition between video games and gambling and highlights promising traits for further and more in-depth qualitative investigations in a complex and constantly evolving matter of gaming and gambling, the risks they pose to mental health and the challenges to specialised treatment offer. Moreover, currently there is no research that confirm the direction of the development of the disorders discussed here. Hence, it is also necessary to conduct longitudinal studies to investigate the influence of gambling features in video games (e.g. loot boxes) on the development of gambling disorders. The problems emphasised here can be informative for various educational or preventive campaigns, especially addressed to young people.

## Limitations

The study presented here does not come without limitations. One of them is the consequences of setting the age of the group of video game players to 25 years old. By doing so, excluded the perspective of older players who could probably indicate other factors that might contribute to the transition from playing video games to gambling. Bias in recruitment of people with gambling disorders would be another limitation. During fieldwork procedure, we asked therapists for their support in recruitment, which may have had an impact on the selection of a particular profile of patients. Another limitation was imposed by COVID-19 sanitary regime rules which limited field research opportunities and made us decide to conduct the interviews by a telephone or using an online platforms with video transmission which could also affect the process of data collection.

## Conclusions

Our research shows that among gamers and individuals with gambling disorder there is an explicit awareness of several similarities between video games and gambling, that might be considered risk factors for switching from games to gambling. Similar emotional states that accompany gamers and gambling players may drive the transition from video games to gambling and therefore should be emphasized in a treatment programmes for people with gambling disorders. Addiction therapists need to pay attention to the fact of playing video games containing loot boxes as that can be associated with the higher risk of developing disorders related to playing games. Moreover, decision-makers should introduce effective regulation of the use of gambling components in video games, e.g. loot boxes or roulettes, as well as implement educational campaigns to increase the awareness of gambling risk among the growing number of individuals who engage in video gaming, and to protect them against the gambling-related harm.

## Data Availability

The data that support the findings of this study are available from the corresponding author upon request.
